# Added Value of Anti-CD74 Autoantibodies in Axial SpondyloArthritis in a Population With Low HLA-B27 Prevalence

**DOI:** 10.3389/fimmu.2019.00574

**Published:** 2019-03-26

**Authors:** Nelly R. Ziade, Iyad Mallak, Georges Merheb, Pierre Ghorra, Niklas Baerlecken, Torsten Witte, Xenofon Baraliakos

**Affiliations:** ^1^Department of Rheumatology, Saint-Joseph University, Beirut, Lebanon; ^2^Department of Rheumatology, Hotel-Dieu de France Hospital, Beirut, Lebanon; ^3^Department of Radiology, Hotel-Dieu de France Hospital, Beirut, Lebanon; ^4^Department of Rheumatology, Holy Spirit University, Kaslik, Lebanon; ^5^Department of Rheumatology, ND Secours Hospital, Byblos, Lebanon; ^6^Blood Transfusion Center, Hotel-Dieu de France Hospital, Beirut, Lebanon; ^7^Private Practice Rheumatology, Cologne, Germany; ^8^Department of Immunology and Rheumatology, Medical University, Hanover, Germany; ^9^Rheumazentrum Ruhrgebiet, Herne, Germany; ^10^Ruhr-University Bochum, Bochum, Germany

**Keywords:** spondyloarthritis, autoantibodies, autoimmunity, diagnosis, HLA-B27, anti-CD74 antibodies

## Abstract

Axial spondyloarthritis (axSpA) is often diagnosed late due to the non-specific nature of its main symptom [chronic back pain (CBP)] and to the paucity of diagnostic markers, particularly in regions with low HLA-B27 prevalence, such as the Middle-East. We tested the performance of IgG4 and IgA anti-CD74 antibodies as an early diagnostic marker for axSpA, compared with the performance of HLA-B27, in Lebanon. Sera of axSpA patients diagnosed by the rheumatologist and also fulfilling the imaging arm of the ASAS criteria (patients) and of blood donors (BD) (controls) were analyzed for HLA-B27, IgG4 and IgA anti-CD74, blinded to clinical characteristics. Receiver Operating Characteristic curves were constructed to identify an optimal cut-off point for anti-CD74 antibodies. Diagnostic properties were calculated (sensitivity, specificity, positive, and positive predictive values (PPV, NPV), Likelihood ratios) for each marker. Forty-nine axSpA patients and 102 BD were included in the final analysis. IgA anti-CD74 correlated poorly with axSpA (Area Under the Curve (AUC) 0.657), whereas IgG4 anti-CD74 had a good discriminative value (AUC 0.837). Respectively, for HLA-B27, IgG4 anti-CD74, and the combination of both, we found a sensitivity of 33-92-33%, specificity of 96-79-98%, PPV 80-68-89%, NPV 75-95-75%, and LR+ 8.2-4.4-16.5. IgG4 anti-CD 74 were positive in 88% of HLA-B27 negative axSpA patients, and correlated with BASDAI. In this first study in a population with low HLA-B27 prevalence, IgG4 anti-CD74 antibodies combined with HLA-B27 showed higher diagnostic value than HLA-B27 alone for early axSpA. IgG4 anti-CD74 should be considered for further evaluation as an early axSpA diagnostic marker in future dedicated research, particularly in patients with CBP.

## Introduction

Axial spondyloarthritis (axSpA) is often diagnosed late, due to the non-specific nature of its main symptom, chronic back pain (CBP) and to the paucity of diagnostic markers, and its pathogenesis is still unclear (1–5). Recent studies showed that early diagnosis leads to a better prognosis, supporting the strong need for reliable diagnostic biomarkers (6–9).

Classification criteria serve as surrogates for diagnosis in clinical practice and rely on CBP associated with either sacroiliitis on imaging or positive Human Leukocyte Antigen-B27 (HLA-B27), in addition to other clinical or biological parameters (10, 11). Although MRI has allowed a significant reduction in the diagnosis gap, recent studies found “positive MRI-sacroiliitis” in normal individuals, thus challenging the accuracy of the current imaging definition (12, 13). Diagnosis is particularly challenging in the Middle East region (14), where HLA-B27 is reported to be lower than in European countries (15) and the need for other disease markers is particularly crucial.

Non-HLA genetic factors contributing to axSpA susceptibility were described (16) but their value in clinical practice is not determined yet.

A strong association between autoantibodies against CD74 (anti-CD74, Cluster of Differentiation 74) and radiographic axSpA (r-axSpA) was identified in two preliminary studies published in 2013 (17, 18). CD74 was shown to be involved in the assembly of major histocompatibility complex II and in preventing premature binding of peptides to it and was described as a potential factor in the pathogenesis of axSpA, with a possible role in bone formation (19–22). In patients with known r-axSpA, sensitivity of anti-CD74 for r-axSpA was 85.1%, specificity was 92.2%, positive likelihood ratio (LR+) was 10.8 and negative likelihood ratio (LR-) was 0.08. Interestingly, 76.4% of patients were HLA-B27 positive. This association was recently studied in European patients with inflammatory back pain of ≤ 2 years duration and clinical suspicion of axSpA and found a sensitivity of Immunoglobulin A (Ig A) anti-CD74 of 47%, a specificity of 95.3% and a LR+ of 10.0 (International SpondyloArthritis autoantibody trial -InterSpA-) (23). The combination of anti-CD74 and HLA-B27 was particularly interesting, with a post-test probability of 80.2% [assuming a 5% pre-test probability of axSpA in CBP (24, 25)], while being of 33.3% for IgA anti-CD74 alone and 28.8% for HLA-B27 alone. In another European cohort (26), IgA anti-CD74 were present in 54.7% of axSpA patients and 46.2% of CBP patients while IgG anti-CD74 were present in 46.4% of axSpA patients and 37% of CBP patients (26). However, the distinction between axSpA and CBP in this study may not have been perfect in this study, as many CBP patients were HLA-B27 positive.

To date, the diagnostic properties of anti-CD74 have never been tested in populations with low HLA-B27 genetic prevalence, where it could potentially be of high additional value.

In this study (InterSpA-Lebanon), we tested the performance of IgG4 and IgA anti-CD74 as an early diagnostic markers for axSpA compared with the performance of HLA-B27 in Lebanon, which is known as one of the countries with the lowest HLA-B27 prevalence ever reported (15).

## Materials and Methods

The patients were recruited from primary and specialized rheumatology clinics across the Lebanese territory, after a vast advertising program among rheumatologists and primary care physicians.

Inclusion criteria were: age between 18 and 45 years at study visit, Lebanese origin, short disease duration (>3 years duration), axSpA diagnosed by the rheumatologist, and also fulfilling the Assessment of SpondyloArthritis international Society (ASAS) criteria imaging arm (11), active sacroiliitis on MRI (as per ASAS criteria) (10).

Exclusion criteria were other inflammatory rheumatic diseases, prior exposure to biologic therapy, corticosteroids ≥10 mg prednisolone/day for ≥4 weeks, contra-indication to MRI (claustrophobia, gadolinium intolerance), pregnancy and lactation.

Blood donors (BD) were recruited from one center which receives donors from all over the Lebanese territory; they were matched for demographics.

Patients and BD gave informed consent for use of their sera in the study, and their basic demographic data were collected. The study was approved by the local Ethics Committee of Saint-Joseph University, which acts in line with the rules of Good Clinical Practice described in the Helsinki declaration (October 2013 version).

Clinical data and sera sample collection were performed in one visit at a single centralized university center. Demographic information including patients' age, gender and region of origin were collected in axSpA patients and BD. Disease characteristics including the site and features of central and peripheral pain, articular and extra-articular manifestations [psoriasis, peripheral arthritis, enthesitis, uveitis, dactylitis, inflammatory bowel diseases (IBD)], family history of SpA, psoriasis, uveitis, infections and IBD, response to medication (Non-Steroidal Anti-Inflammatory Drugs (NSAIDs), analgesics, corticosteroids) were collected. Clinical examination recorded peripheral arthritis, enthesitis, dactylitis, and measures for disease activity such as the Bath Ankylosing Spondylitis Disease Activity Index (BASDAI), Bath Ankylosing Spondylitis patient Global score (BAS-G) and the Ankylosing Spondylitis Disease Activity Score (ASDAS). The spinal mobility was assessed by the Bath Ankylosing Spondylitis Metrology Index (BASMI).

Erythrocyte Sedimentation Rate (ESR) and C-Reactive Protein (CRP) were measured locally. Local HLA-B27 results were collected in this first step, only when previously available.

Radiographic data (pelvis x-rays and sacro-iliac MRI) were sent electronically to a single experienced reader and analyzed blinded to any characteristics to decide upon the final inclusion of the patients in the study and their classification into radiographic (r-axSpA) or non-radiographic (nr-axSpA) axSpA.

DNA of axSpA patients and BD were analyzed centrally for HLA-B27 genes (Polymerase Chain Reaction), without subtype analysis, and sera for IgA and IgG4 antibodies against CD74 by ELISA with specificity for CLIP (Class II-associated invariant chain peptide domain of the CD74 protein consisting of 25 amino-acids) developed in cooperation with AESKU Diagnostics, Germany. The details of testing are described elsewhere (23). All data was anonymized at the local laboratory by attributing a number to each subject upon blood withdrawal and the central laboratory workers were completely blinded for clinical data.

Comparisons between groups were evaluated using the Mann-Whitney *U*-test. For the diagnostic properties of anti-CD74, Receiver Operating Characteristic (ROC) curves were constructed and areas under the curve (AUC) determined, before identifying an optimal cut-off point. Diagnostic properties were calculated (sensitivity, specificity, positive predictive value (PPV), negative predictive value (NPV), LR+ and LR-) for HLA-B27, IgG4, IgA and Immune complexes (IC) of anti-CD74. *T*-test and linear regression were used to identify the association between anti-CD74 and the clinical characteristics. A *p* < 0.05 was regarded as statistically significant. Statistical analysis was performed with SPSS 25.0.

## Results

Sixty patients with axSpA as diagnosed by the rheumatologist were recruited and offered initial clinical examination, blood exam, sacro-iliac x-ray and MRI analysis. Eleven patients were excluded secondarily for the following reasons: axSpA was not confirmed by central reading of images in 6 cases, 2 patients had Armenian close ancestry and therefore were considered as having a non-Lebanese genetic background, 2 patients had concomitant conditions that may have interfered with the antibodies levels (one patient developed positive rheumatoid factor at a subsequent visit and another patient was treated with investigational medicine) and 1 patient had a longer disease duration than 3 years, discovered after referral to the study. Finally, the sera of 49 axSpA patients and 102 BD were analyzed and compared.

AxSpA patients were slightly older than BD (34 and 30 years, respectively, *p* = 0.008) with no other demographic differences. 57.1% of the axSpA population were males (28/49). Fifty-five percent of patients referred with the diagnosis of axSpA were classified as nr-axSpA (27/49). Mean symptoms duration was 25.3 months (SD 16), mean BASDAI was 4.3 (SD 2.1) and mean ASDAS-CRP was 3.4 (SD 1.2) (Table 1). Eighty percent had pain in the buttock area, 92% in the lumbar spine, 14% in the thoracic spine and 27% in the cervical spine. The most common extra-articular manifestations were enthesitis (22%), psoriasis (14%), peripheral arthritis (12%), inflammatory bowel diseases (8%), and uveitis (8%). Eighteen percent had a family history of SpA.

**Table 1:** Baseline characteristics of the axSpA patients.

Total	49
Age in years (*SD*)	34.1 (8.1)
Males	28 (57.1%)
**Place of birth**	
- Beirut (capital)	11 (22.4%)
- Rest of the country	38 (77.6%)
**Disease**	
- r-axSpA	22 (45%)
- nr-axSpA	27 (55%)
Symptom duration (*SD*)	25.3 months (16)
**Pain site**	
- Buttocks	−42 (86%)
- Lumbar spine	−45 (92%)
- Thoracic spine	−7 (14%)
- Cervical spine	−13 (27%)
**Extra-articular manifestations**	
- Enthesitis	−11 (22%)
- Psoriasis	−7 (14%)
- Peripheral arthritis	−5 (12%)
- Uveitis	−4 (8%)
- IBD	−4 (8%)
- Infection	−1 (2%)
Positive family history of SpA	9 (18%)
BASDAI, mean (*SD*)	4.3 (2.1)
ASDAS-CRP, mean (*SD*)	3.4 (1.2)

HLA-B27 status was positive in 16/49 axSpA patients and in 4/102 BD (sensitivity 33%, specificity 96%, Positive Predictive Value (PPV) 80%, Negative Predictive Value (NPV) 75% and LR+ 8.2) (Table 2). Positive association was found between HLA-B27 and place of birth (highest in Beirut, OR 2.26, *p* = 0.014) and a positive family history of SpA (OR 34.75, *p* < 0.001). We found no association of HLA-B27 with other demographic factors, such as gender, nor with disease characteristics. Mean levels of anti-CD74 were significantly higher in axSpA patients compared to BD for IgG4 (1.29 vs. 0.58, *p* = 0.001), for IgA (1.03 vs. 0.73, *p* = 0.034) but not for IgA-Immune complexes (0.61 vs. 0.49, *p* = 0.089).

**Table 2:** Diagnostic properties of HLA-B27 in axSpA compared to Blood Donors (BD).

	**axSpA**	**BD**	**Total**	**Predictive value**
HLA-B27+	16	4	20	PPV 80%
HLA-B27-	33	98	131	NPV 75%
Total	49	102	151	
	Sensitivity 33%	Specificity 96%		LR+ 8.2

**Figure 1:**
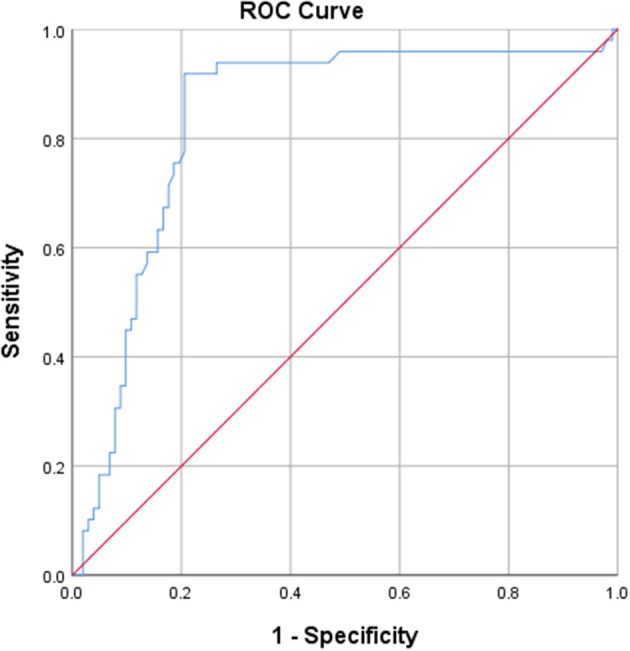
ROC curve derived from IgG4 anti-CD74, showing a good discriminative value for IgG4 anti-CD74.

Calculation of the AUC, with an optimal cut-off of 0.5148, was 0.837 (*p* < 0.001) for IgG4, translating a good discriminative value (Figure 1). The AUC was 0.657 (*p* = 0.045) for IgA, which is considered poorly discriminative (cut-off 1.12).

Using 0.5148 as cut-off, the sensitivity of IgG4 anti-CD74 was 92%, specificity was 79%, PPV was 68%, NPV was 95%, LR+ was 4.4 and LR- was 0.1 (Table 3).

**Table 3:** Diagnostic properties of IgG4 anti-CD74 in axSpA compared to Blood Donors.

	**axSpA**	**BD**	**Total**	**Predictive Value**
IgG4 Anti-CD74+	45	21	66	PPV 68%
IgG4 Anti-CD74-	4	81	85	NPV 95%
Total	49	102	151	
	Sensitivity 92%	Specificity 79%		LR+ 4.4

The only parameter associated with positive IgG4 anti-CD74 was BASDAI (*p* = 0.029) as a continuous variable, mainly in relation with the pain domain. When considering BASDAI as a qualitative binary variable, all patients with high BASDAI (≥4) were positive for IgG4 anti-CD74, whereas 86% of patients with low BASDAI (<4) were positive for IgG4 anti-CD74 (*p* = 0.578).

We found no association with demographic (particularly gender), clinical, biological parameters, nor there was a difference in IgG4 anti-Cd74 between radiographic and non-radiographic axSpA (*p* = 0.82).

When considering the axSpA HLA-B27 negative subpopulation (Table 4), IgG4 anti-CD74 were positive in 29 additional /33 axSpA patients (87.9%). The correlation between the two markers was not statistically significant (*p* = 0.193).

**Table 4:** IgG4 anti-CD74 results according to HLA-B27 status in axSpA patients.

	**HLA-B27+**	**HLA-B27-**	**Total**
IgG4 Anti-CD74+	16	**29**	45
IgG4 Anti-CD74-	0	4	4
Total	16	33	49

Bold value indicates high clinical significance.

The combination of HLA-B27 and IgG4 anti-CD74 increased specificity to 98% (with 2 blood donors out of 4 being correctly classified as negative), with a substantial increase in LR+ to 16.5 (Table 5).

**Table 5:** Diagnostic value of the combination of HLA-B27 and IgG4 anti-CD74 in axSpA.

	**axSpA**	**BD**	**Total**	**Predictive Value**
HLA-B27+ AND IgG4 Anti-CD74+	16	2	18	PPV 89%
HLA-B27 – OR IgG4 Anti-CD74-	33	100	133	NPV75 %
Total	49	102	151	
	Sensitivity 33%	Specificity 98%		LR+ 16.5

The different scenarios of diagnostic properties (HLA-B27 alone, IgG4 anti-CD74 alone and the combination of both) are summarized in Table 6.

**Table 6:** Comparative diagnostic value of HLA-B27 alone, IgG4 anti-CD74 alone and combinations for ax SpA.

	**Sensitivity**	**Specificity**	**PPV**	**NPV**	**LR+**
HLA-B27+	33	96	80	75	8.2
IgG4 anti-CD74 +	92	79	68	95	4.4
HLA-B27 + AND IgG4 Anti-CD74 +	33	98	89	75	16.5

## Discussion

The InterSpA–Lebanon trial showed that IgG4 anti-CD74 have good diagnostic properties in Lebanese patients with early axSpA as diagnosed by the rheumatologist and fulfilling the imaging arm of the ASAS classification criteria, with a sensitivity of 92%, specificity of 79% and a LR+ of 4.4.

In addition, our study confirmed the low prevalence of HLA-B27 in the Lebanese axSpA patients (33%) and general population (4%), which was previously suggested by a nationwide study in 1997 (27), but nevertheless showed an excellent specificity (96%) and a high LR+ (8.2) for this established marker.

Each marker alone may not be highly reliable; however, the combination of both, compared with HLA-B27 alone, improved specificity to 98%, with a substantial increase of LR+ to 16.5. This would translate into a considerable increase in the post-test probability of axSpA to 82.5% when considering a background pre-test probability of 5% in a population of CBP. The high added value of this combination is mostly due to the identification of IgG4 anti-CD74 in 88% of HLA-B27 negative patients. As in previous studies (23), anti-CD74 were not correlated with HLA-B27, which highlights the diagnostic value of this new marker, and may provide additional clues for a different pathogenic pathway.

Compared to the InterSpA European trial, the axSpA Lebanese population was slightly older (34 vs. 29 years), with a longer disease duration (25 vs. 12.5 months) and lower -as expected- HLA-B27 prevalence (33 vs. 81%). The male/female ratio was 1.33 in our study (quite similar to 1.27 in InterSpA (23). Forty-five percent of our patients had r-axSpA (vs. 0%) and 100% had positive sacroiliitis according to the ASAS definition (vs. 81%) due to slight differences in the inclusion criteria. Gender balance (57 vs. 56%) and BASDAI (4.3 vs. 4.2) were similar. The diagnostic properties of anti-CD74 seem to be better in our population, particularly when combined with HLA-B27. However, a major difference was observed regarding the type of Ig: a positive correlation with IgG4 was found in our study whereas the higher correlation was with IgA in the European InterSpA trial. This difference limits the direct comparison between the results but may interestingly provide supplementary clues for the role of anti-CD74 in the pathogenesis of axSpA. Since IgA is found in high concentration in the mucous membranes, particularly lining the gastro-intestinal tract, the Ig subtype may reflect the potential impact of an interaction with the gut microbiome on disease incidence and phenotype in different populations.

Our study also identified an association of IgG4 anti-CD74 with BASDAI, which may be an indicator of a possible role of this new marker for the prediction of high disease activity; this finding might be of interest for future investigation in this direction.

Our study has some limitations. First, we determined the positivity threshold in our local population, based on the ROC results, instead of using the manufacturer's positivity threshold. However, it is generally recommended for ELISA tests to establish local cut-offs in every population, especially that the manufacturer's cut-off was developed with sera from German blood donors. Second, our study did not include a CBP arm, which is the target population where the test will need to be applied in practice. In fact, our study was designed and conducted before the publication of the results of the European trials that showed high levels of IgA anti-CD74 in CBP (23, 26), and it remains a pilot study whose results should be confirmed further. Third, the pre-test probability of 5% was derived from European studies, since similar data is not available in our region. Fourth, the relatively small sample size and the small number of individuals with HLA-B27 may be responsible of some fluctuations in the calculated likelihood ratios.

Nevertheless, despite these limitations, our study is a pioneer trial that clearly identified an added value of a new diagnostic marker that can be useful for the early diagnosis of axSpA, particularly in a population with low HLA-B27 prevalence. The improvement in the diagnostic algorithm is expected to shorten the delay between symptoms onset and diagnosis, therefore having a positive impact on the disease course and prognosis.

Should the added value of IgG4 anti-CD74 be confirmed in further studies, cost-effectiveness analysis would determine whether it should be performed simultaneously with HLA-B27 as a first line diagnostic test in patients with a suspicion of axSpA, or if a two-step diagnostic algorithm would be recommended.

## Conclusions

In this first study in a population with low HLA-B27 prevalence, IgG4 anti-CD74 antibodies combined with HLA-B27 showed higher diagnostic value than HLA-B27 alone for early axSpA. Our results indicate that IgG4 anti-CD74 should be considered for further evaluation as an early axSpA diagnostic marker in future dedicated research.

## Data Availability

The datasets generated for this study are available on request to the corresponding author.

## Ethics Statement

This study was carried out in accordance with the recommendations of the Ethics Committee of the Saint-Joseph University, Beirut, Lebanon, with written informed consent from all subjects. All subjects gave written informed consent in accordance with the Declaration of Helsinki. The protocol was approved by the Ethics Committee of the Saint-Joseph University, Beirut, Lebanon.

## Author Contributions

NZ, TW, and XB designed the study, supervised data collection, analyzed the results, and participated in the manuscript writing. IM collected the clinical data, supervised blood sample packaging, performed data entries, and participated in the manuscript review. TW and NB analyzed and provided central laboratory results, as well as manuscript details about central laboratory results. GM participated in the data collection and manuscript review. PG analyzed and provided central laboratory results, supervised blood samples transportation from local to central lab, and participated in the manuscript review. NZ supervised all field work on site, conducted the analysis and manuscript design. XB provided central reading of all x-rays and MRIs. All authors reviewed the manuscript and provided comments on the article.

### Conflict of Interest Statement

This study is funded by AbbVie Germany. AbbVie contributes to study logistics and site management. The authors determined study design, conduct of the study and the content of the publication. No payments were made to the authors for writing this manuscript. NB and TW share a patent on the commercial use of antibodies against CD74, which will in the future be produced by Aesku Diagnostics (Eva Schweikhard and Torsten Matthias). The remaining authors declare that the research was conducted in the absence of any commercial or financial relationships that could be construed as a potential conflict of interest.
